# Prediction model for prolonged fever in patients with *Mycoplasma pneumoniae* pneumonia: a retrospective study of 716 pediatric patients

**DOI:** 10.1186/s12890-021-01534-2

**Published:** 2021-05-18

**Authors:** Min Sik Jang, Bit Gyeol Kim, Jihye Kim

**Affiliations:** Department of Pediatrics, Hallym University College of Medicine, Kangdong Sacred Heart Hospital, 150, Seongan-ro, Gangdong-gu, Seoul, 05355 Republic of Korea

**Keywords:** *Mycoplasma pneumoniae* pneumonia, Refractory, Prediction model

## Abstract

**Objective:**

To identify patients with *Mycoplasma pneumoniae* pneumonia (MPP) with a risk of prolonged fever while on macrolides.

**Methods:**

A retrospective study was performed with 716 children admitted for MPP. Refractory MPP (RMPP-3) was defined as fever persisting for > 72 h without improvement in clinical and radiologic findings after macrolide antibiotics (RMPP-3) or when fever persisted for > 120 h (RMPP-5) without improvement in clinical and radiologic findings. Radiological data, laboratory data, and fever profiles were compared between the RMPP and non-RMPP groups. Fever profiles included the highest temperature, lowest temperature, and frequency of fever. Prediction models for RMPP were created using the logistic regression method and deep neural network. Their predictive values were compared using receiver operating characteristic curves.

**Results:**

Overall, 716 patients were randomly divided into two groups: training and test cohorts for both RMPP-3 and RMPP-5. For the prediction of RMPP-3, a conventional logistic model with radiologic grouping showed increased sensitivity (63.3%) than the model using laboratory values. Adding laboratory values in the prediction model using radiologic grouping did not contribute to a meaningful increase in sensitivity (64.6%). For the prediction of RMPP-5, laboratory values or radiologic grouping showed lower sensitivities ranging from 12.9 to 16.1%. However, prediction models using predefined fever profiles showed significantly increased sensitivity for predicting RMPP-5, and neural network models using 12 sequential fever data showed a greatly increased sensitivity (64.5%).

**Conclusion:**

RMPP-5 could not be effectively predicted using initial laboratory and radiologic data, which were previously reported to be predictive. Further studies using advanced mathematical models, based on large-sized easily accessible clinical data, are anticipated for predicting RMPP.

## Background

*Mycoplasma pneumoniae* (MP) infections are generally mild and self-limiting. However, patients of every age can develop a severe and progressive course during treatment with appropriate antibiotic therapy [1]. The underlying mechanisms are unclear, but a direct microbe effect, macrolide resistance, and excessive immunological response of the host are commonly suggested [2, 3]. Macrolide antibiotics have been generally preferred as the first-choice agents for MP infections because secondary antibiotics such as tetracyclines and fluoroquinolones are not recommended because of the risk of severe adverse events, especially in pediatric patients.

Macrolide resistance rates have risen throughout the world and vary across countries[4–7]. Although macrolides could be continued in cases of mild to moderate infections irrespective of their resistance, replacement by alternative antibiotics or additional corticosteroids have been shown to improve radiological abnormalities and clinical symptoms[8, 9]. Additionally, the severity of the disease is partially related to the degree to which the host immune response reacts to infection. The concept of immune-mediated lung disease provides a basis for consideration of immunomodulatory therapy in addition to conventional antimicrobial therapies for the management of MP infections [10].

The appropriate time for alternative treatment is not clarified, but it still depends on the physician’s decision. Alternative treatments are delayed on some occasions owing to concerns regarding toxicities and adverse effects of secondary antibiotics or the possibility of blurred diagnosis caused by corticosteroids, leading to aggravation of the clinical course. Protracted courses of fever or worsening respiratory exertion despite treatment with macrolides are reported to complicate atelectasis, parapneumonic effusion, bronchiolitis obliterans, necrotizing pneumonitis, pulmonary abscess, and systemic inflammatory response syndrome [2, 11–14]. At the initiation phase of macrolide therapy, physicians find it difficult to predict patients with a prolonged or severe clinical course. Previous studies have suggested individual cut-off values for inflammatory markers to differentiate between the patients with or without clinical and radiological progression after macrolide therapy for 7 days or longer [8, 15–20]. Identifying patients who are expected to undergo a prolonged or severe clinical course would help in providing them with timely secondary treatment and mitigating their clinical course [8, 17, 21, 22].

This study aims to identify the predictive factors for prolonged fever in patients with MP pneumonia with readily accessible clinical, laboratory, and radiological data and to develop a predictive model for these patients in whom timely initiation of secondary treatment options should be considered.

## Methods

### Study design and ethical considerations

The medical records of previously healthy children admitted for MP infection at our institution between January 2015 and December 2019 were retrospectively reviewed. The study was designed and conducted using the format recommended by the Strengthening the Reporting of Observational Studies in Epidemiology guidelines. The study protocol was approved by the Institutional Review Board of Kangdong Sacred Heart Hospital. The review board waived the requirement for informed consent for this study.

### Study patients

All patients who had symptoms and signs indicative of pneumonia at admission, including fever (≥ 38 °C), cough, and abnormal lung auscultation, were included. Empiric antibiotics were initially prescribed for these patients (β-lactam agents and/or macrolides). Only patients initiated on a regimen with macrolides were included. When the patients were considered to have persistent fever with no improvement in their clinical status and radiologic findings after 72 h or longer of macrolide treatment, they were either continued on macrolides, started on additional intravenous methylprednisolone (1–2 mg/kg/d for 3–5 days), with or without addition of secondary antibiotics (tetracyclines or fluoroquinolones), depending on their clinical, laboratory, and radiologic findings.

Diagnosis of *M. pneumoniae* pneumonia was confirmed by laboratory data and chest radiographs. A baseline blood sample and nasopharyngeal aspirate/swab (NPA) were collected for serological and microbiological testing. *M. pneumoniae* infection was confirmed using serologic testing and/or polymerase chain reaction (PCR) testing of the NPA. An enzyme immunoassay for IgM antibodies specific to *M. pneumoniae* (EIA, Bio-Rad Platelia™ *M. pneumoniae* IgM, California, USA) was performed with the initial blood samples according to the manufacturer’s protocol. MP infection was confirmed when a positive IgM titer and/or a positive PCR result for *M. pneumoniae* was observed. When the initial result of IgM antibodies was negative in a highly suspected patient without a positive PCR result, it was then repeated every 2–3 days thereafter until a positive conversion was confirmed to avoid missing false-negative cases.

We excluded patients with underlying diseases, patients who were treated for confirmed or suspected MP infection within the prior four weeks, patients with either positive IgM or PCR for MP but whose symptoms and radiographic findings were incompatible with pneumonia, patients treated with antiviral agents for proven influenza virus with fever onset within 72 h, patients who received intravenous corticosteroids or were changed to alternative antimicrobials (tetracyclines or fluoroquinolones) within 72 h, and patients who were afebrile after admission. Although some of the patients had received additional treatment including intravenous methylprednisolone or secondary antibiotics after 72 h, only those patients whose fever and clinical symptoms persisted longer than 120 h after the additional treatment were included in the cohort.

### Definitions of RMPP-3 and RMPP-5

A case with persistent fever for > 72 h without improvement in the clinical and radiological findings despite appropriate management with macrolides was defined as refractory *M. pneumoniae* pneumonia (RMPP-3). Patients with persistent fever for > 120 h without improvement in the clinical and radiological findings despite appropriate management were defined as RMPP-5. We hypothesized that the predictive variables for fever > 72 h and fever > 120 h would differ. We targeted to identify and compare those variables within the same cohort which was alternatively divided into training and test cohorts by these two definitions.

### Grouping: training and test cohorts for RMPP-3 and RMPP-5

**Fig. 1:**
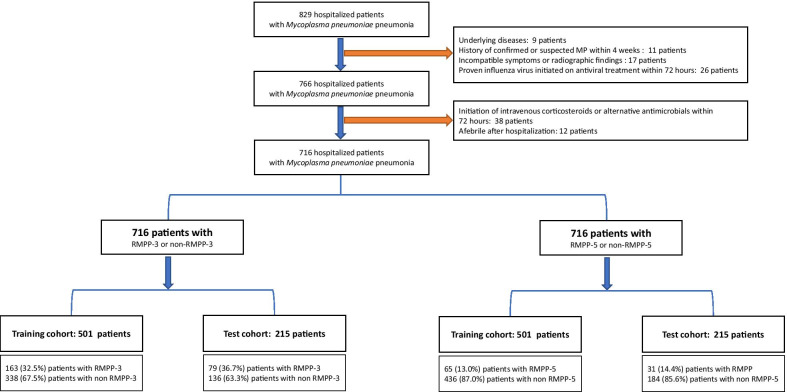
Study flow diagram

Patients were randomly grouped into the training (n = 501) and test cohorts (n = 215) by 67:33 splitting using the Python Scikit-learn library (Fig. 1). Each cohort was then categorized into the RMPP-3 group and non-RMPP-3 group based on their duration to defervescence. Defervescence was defined as maintenance of body temperature below 38 °C for at least 24 h. For the prediction analysis of patients with fever for > 120 h, the group randomization process was implemented again on the same cohort, after which each cohort was categorized into the RMPP-5 and non-RMPP-5 groups.

### Predictors: fever profiles

The frequency of fever was defined as the number of peaks on the temperature curve. It was only counted when body temperature was ≥ 38.0 °C and had increased ≥ 0.6 °C within 4 h. If the patient continued to have temperature changes of< 0.6 °C but whose body temperature was ≥ 38.0 °C during the 4-h interval, it was counted as valid (continuous fever pattern).

### Predictors: clinical data

Demographic and clinical information were collected in a standardized form by reviewing the electronic medical records. The following information was gathered: duration of fever (before and after hospitalization), total hospital days, and fever profile (highest body temperature, lowest body temperature, frequency of peak fever over 39 °C, frequency of peak fever over 40 °C, and total frequency of peak fever) extracted from 12 sequential fever data within 48 h. These fever profiles were only included in the analysis for the prediction of prolonged fever over 120 h (RMPP-5).

### Predictors: laboratory data

Tests for complete blood count (CBC), serum aminotransferase, erythrocyte sedimentation rate (ESR), C-reactive protein (CRP), lactate dehydrogenase (LDH), procalcitonin, and blood cultures were performed. FilmArray multiplex PCR system (Biomérieux, USA) was used for detecting common respiratory tract virus antigens.

### Predictors: radiologic data

Chest radiographs were reviewed independently by two experienced radiologists. They were blinded to the clinical data and original radiographic interpretations. Radiological findings at admission were categorized into four groups: group 1, patients with parahilar peribronchial opacification or diffuse interstitial infiltration; group 2, patients with reticular, nodular, or reticulonodular densities; group 3, patients with segmental or lobar consolidation in a single lobe with or without pleural effusion of 1/4–1/2 in the decubitus position; and group 4, patients with lobar consolidation in 2 or more lobes and/or pleural effusion of more than 1/2 in the decubitus position. The images were interpreted and compared by two radiologists to reach a consensus.

### Statistical analyses

Continuous variables were presented as mean ± standard deviation and were compared using an independent *t-*test. Categorical variables were presented as frequency (%) and were compared using the Pearson chi-squared test or Fisher’s exact test.

Based on the data from the training cohorts, the univariate logistic regression analysis was performed for identifying significant independent predictors for RMPP-3 or RMPP-5. With the significant predictors, stepwise multivariate logistic regression analysis was performed for creating conventional prediction models. To reflect the 12 sequential fever data on the prediction models effectively, a deep neural network (DNN) model was additionally created. DNN included two hidden layers. A dropout layer was used after the first hidden layer to prevent overfitting. For hyperparameter optimization, 20% of the training cohort patients were assigned to the validation cohort. Optimization was performed using the Adam method, and model loss was calculated through binary cross-entropy. Calculations to determine the optimal number of layers and neurons for all DNNs were performed. For each combination of layers and hidden units, hyperparameters for obtaining the best performance for the combination were optimized.

The prediction power of the conventional logistic prediction model and the DNN model was evaluated in the test cohorts using receiver operating characteristic (ROC) curves. Logistic regression analysis was performed using SPSS 25 (IBM Corp., Armonk, NY, USA). DNN models were developed using Python 3.7 (open-source projects) with Anaconda 4.7.12, and TensorFlow 2.0.

## Results

### Baseline characteristics

Overall, 716 patients with *M. pneumoniae* were enrolled during the five-year study period after applying the exclusion criteria. The mean age of the entire cohort was 5.6 years (range, 1–16 years), and 350 patients (48.8%) were boys. No patients were transferred to the intensive care unit or received mechanical ventilation. Doxycycline and intravenous levofloxacin were finally prescribed in 36 patients (5.0%) and 10 patients (1.4%), respectively. One hundred sixty-three patients (32.5%) in the training cohort (n = 501) and 79 patients (36.7%) in the test cohort (n = 215) were classified as RMPP-3 (Fig. 1). Sixty-five patients (13.0%) in the training cohort and 31 patients (14.4%) in the test cohort were classified as RMPP-5.

In the training cohort for RMPP-3, duration of fever at admission were not significantly different between the RMPP group and non-RMPP group (*p* = 0.057). Duration of fever after admission and the total duration of hospitalization were significantly longer in the RMPP-3 group (*p*< 0.001) than in the non-RMPP-3 group (Table 1). In the training cohort for RMPP-5, however, fever duration at admission was longer in the non-RMPP-5 group (*p*< 0.001) compared with RMPP-5 group (Table 2). No difference was observed in the rates of concurrent respiratory virus detection between RMPP group and the non-RMPP group.

**Table 1:** Initial variables of the whole cohort (fever > 72 h)

	Training cohort (*n* = 501)			Test cohort (*n* = 215)		
	Fever ≥ 72 h	Fever < 72 h	*p*-value	Fever ≥ 72 h	Fever < 72 h	*p*-value
Number of patients (*n,* %)	163 (32.5)	338 (67.5)		79 (36.7)	136 (63.3)	
Age, mean (years)	5.9 ± 2.8	5.5 ± 3.2	0.140	5.8 ± 2.5	5.3 ± 3.1	0.273
Sex ratio (female: male)	1.3	1.0	0.124	1.7	0.7	0.002
Duration of fever (days)						
At admission	4.8 ± 1.8	5.1 ± 2.2	0.057	4.9 ± 1.7	4.9 ± 2.0	0.960
After admission	4.8 ± 3.4	1.3 ± 1.0	< 0.001	4.9 ± 3.3	1.3 ± 0.9	< 0.001
Total	9.4 ± 3.3	6.5 ± 2.3	< 0.001	9.5 ± 3.0	6.3 ± 2.1	< 0.001
Total hospital days (days)	9.2 ± 3.5	5.4 ± 1.6	< 0.001	9.4 ± 3.6	5.5 ± 1.8	< 0.001
Initial inflammatory markers						
WBC × 10^3^/µL	7.6 ± 3.5	9.6 ± 7.0	0.001	7.5 ± 3.2	9.7 ± 5.5	< 0.001
Neutrophils (%)	67.0 ± 10.7	63.0 ± 14.2	0.001	68.0 ± 10.8	61.9 ± 14.2	0.001
Absolute neutrophil count × 10^3^/µL	5.2 ± 2.9	6.2 ± 5.3	0.003	5.1 ± 2.4	6.2 ± 4.8	0.031
Lymphocytes (%)	23.2 ± 8.4	26.6 ± 12.4	< 0.001	22.9 ± 8.4	27.7 ± 12.8	0.001
Absolute lymphocyte count × 10^3^/µL	1.1 ± 0.5	1.4 ± 1.1	< 0.001	1.1 ± 0.6	1.4 ± 0.7	0.001
Platelet × 10^3^/µL	242.0 ± 75.9	304.9 ± 100.4	< 0.001	234.9 ± 71.1	308.6 ± 101.1	< 0.001
ESR (mm/hr)	38.3 ± 18.4	35.9 ± 18.5	0.178	37.4 ± 17.3	32.5 ± 17.4	< 0.001
CRP (mg/L)	49.2 ± 45.6	24.7 ± 30.6	< 0.001	57.7 ± 53.9	24.1 ± 27.7	< 0.001
Procalcitonin (ng/mL)	0.6 ± 1.2	0.7 ± 2.4	0.700	0.5 ± 0.7	0.3 ± 0.6	0.169
Other laboratory data						
LDH (IU/L)	359.8 ± 107.4	326.2 ± 73.1	< 0.001	372.6 ± 132.8	332.7 ± 90.7	0.019
AST (IU/L)	40.3 ± 17.9	36.2 ± 29.3	0.104	39.4 ± 14.9	41.5 ± 48.5	0.701
ALT (IU/L)	19.6 ± 13.3	22.6 ± 64.9	0.556	15.9 ± 7.3	29.2 ± 77.9	0.051
Concurrent respiratory virus (*n*, %)	36/140 (25.7)	95/285 (33.3)	0.110	18/64 (28.1)	39/117 (33.3)	0.471
Oxygen requirement (*n*, %)	5 (3.1)	8 (2.4)	0.765	3 (3.8)	2 (1.5)	0.359
Radiologic grouping (*n*, %)						
Group 1	13 (8.0)	108 (32.0)	< 0.001	7 (8.9)	44 (32.4)	< 0.001
Group 2	54 (33.1)	161 (47.6)		22 (27.8)	67 (49.3)	
Group 3	85 (52.1)	68 (20.1)		41 (51.9)	24 (17.6)	
Group 4	11 (6.7)	1 (0.3)		9 (11.4)	1 (0.7)	
Pleural effusion (*n*, %)	34 (20.9)	20 (5.9)	< 0.001	18 (22.8)	6 (4.4)	< 0.001
Radiologic aggravation on the 3rd or 4th hospital day (*n*, %)	121 (74.2)	29 (8.6)	< 0.001	61 (77.2)	15 (11.0)	< 0.001

*ESR* erythrocyte sedimentation rate, *CRP* C-reactive protein, *LDH* lactate dehydrogenase, *AST* aspartate aminotransferase, *ALT* alanine aminotransferase, *WBC* white blood cell

Continuous variables are presented as mean ± standard deviation

Categorical variables are presented as frequencies (%)

**Table 2:** Initial variables of the whole cohort (fever > 120 h)

	Training cohort (*n* = 501)			Test cohort (*n* = 215)		
	Fever ≥ 120 h	Fever < 120 h	*p*-value	Fever ≥ 120 h	Fever < 120 h	*p*-value
Number of patients (*n,* %)	65 (13.0)	436 (87.0)		31 (14.4)	184 (85.6)	
Age, mean (years)	6.1 ± 2.5	5.7 ± 3.1	0.217	5.7 ± 3.0	5.3 ± 3.1	0.496
Sex ratio (female: male)	1.8	0.9	0.011	2.4	1.0	0.030
Duration of fever (days)						
At admission	4.2 ± 1.4	5.1 ± 2.1	< 0.001	4.4 ± 1.5	4.9 ± 2.0	0.130
After admission	7.6 ± 3.6	1.7 ± 1.2	< 0.001	7.8 ± 4.1	1.6 ± 1.2	< 0.001
Total	11.0 ± 3.8	7.0 ± 2.3	< 0.001	11.3 ± 4.6	6.6 ± 2.3	< 0.001
Total hospital days (days)	11.8 ± 3.7	6.0 ± 2.0	< 0.001	12.0 ± 4.2	5.7 ± 1.7	< 0.001
Initial inflammatory markers						
WBC × 10^3^/µL	7.5 ± 3.4	9.4 ± 6.8	0.030	7.0 ± 3.0	8.6 ± 3.7	0.031
Neutrophils (%)	71.0 ± 9.6	64.0 ± 13.7	< 0.001	66.8 ± 12.1	62.0 ± 13.0	0.058
Absolute neutrophil count × 10^3^/µL	5.5 ± 3.0	6.2 ± 5.2	0.290	4.5 ± 1.5	5.5 ± 3.1	0.011
Lymphocytes (%)	20.4 ± 7.2	25.8 ± 11.8	< 0.001	23.9 ± 10.0	27.5 ± 11.4	0.106
Absolute lymphocyte count × 10^3^/µL	1.0 ± 0.4	1.3 ± 1.0	< 0.001	1.0 ± 0.5	1.3 ± 0.6	0.020
Platelet × 10^3^/µL	208.2 ± 50.0	298.2 ± 100.9	< 0.001	219.3 ± 72.9	286.3 ± 89.9	< 0.001
ESR (mm/hr)	34.2 ± 15.8	36.2 ± 18.5	0.392	32.8 ± 15.6	36.5 ± 18.9	0.303
CRP (mg/L)	68.4 ± 67.3	29.6 ± 33.9	< 0.001	57.9 ± 53.1	27.7 ± 25.9	0.004
Procalcitonin (ng/mL)	0.7 ± 1.4	0.5 ± 1.8	0.512	1.3 ± 2.3	0.5 ± 1.8	0.261
Other laboratory data						
LDH (IU/L)	379.0 ± 147.6	334.4 ± 85.5	0.020	407.3 ± 139.9	328.9 ± 71.5	0.005
AST (IU/L)	43.1 ± 22.2	38.7 ± 37.3	0.346	46.2 ± 23.1	35.1 ± 11.2	0.013
ALT (IU/L)	17.4 ± 11.6	25.8 ± 71.6	0.351	23.2 ± 16.6	16.2 ± 10.9	0.030
Concurrent respiratory virus (*n*, %)	13 (24.1)	125 (33.7)	0.158	7 (39.2)	43 (27.4)	0.856
Oxygen requirement (*n*, %)	4 (6.2)	9 (2.1)	0.075	2 (6.5)	3 (1.6)	0.152
Radiologic grouping (*n*, %)						
Group 1	3 (4.6)	112 (25.7)	< 0.001	3 (9.7)	54 (29.3)	< 0.001
Group 2	18 (27.7)	194 (44.5)		6 (19.4)	86 (46.7)	
Group 3	33 (50.8)	126 (28.9)		17 (54.8)	42 (22.8)	
Group 4	11 (16.9)	4 (0.9)		5 (16.1)	2 (1.1)	
Pleural effusion (*n*, %)	19 (29.2)	34 (7.8)	< 0.001	9 (29.0)	16 (8.7)	0.003
Radiologic aggravation on the 3rd or 4th hospital day (*n*, %)	59 (90.8)	108 (24.8)	< 0.001	25 (80.6)	34 (18.5)	< 0.001
Fever profiles						
Highest temperature (°C)	39.6 ± 0.6	38.7 ± 0.7	< 0.001	39.4 ± 0.6	38.7 ± 0.7	< 0.001
Lowest temperature (°C)	37.0 ± 0.3	36.6 ± 0.3	< 0.001	36.9 ± 0.3	36.6 ± 0.3	< 0.001
Frequency of fever > 39 °C within 48 h (n)	3.3 ± 2.8	0.7 ± 1.4	< 0.001	2.9 ± 1.7	0.6 ± 1.2	< 0.001
Frequency of fever > 40 °C within 48 h (n)	0.5 ± 1.0	0.1 ± 0.3	< 0.001	0.3 ± 0.6	0.0 ± 0.2	0.070
Frequency of peak fever within 24 h (n)	4.7 ± 1.2	2.9 ± 1.4	< 0.001	4.6 ± 1.0	2.8 ± 1.3	< 0.001
Frequency of peak fever within 48 h (n)	8.7 ± 1.7	4.6 ± 2.7	< 0.001	8.6 ± 1.7	4.5 ± 2.6	< 0.001
12 sequential body temperatures						
Initial (°C)	38.6 ± 0.9	37.9 ± 0.9	< 0.001	38.2 ± 0.9	37.9 ± 0.9	0.100
After 4 h (°C)	38.1 ± 0.9	37.6 ± 0.8	< 0.001	38.0 ± 0.9	37.6 ± 0.7	0.008
After 8 h (°C)	37.9 ± 0.8	37.4 ± 0.9	< 0.001	38.0 ± 1.1	37.5 ± 0.8	0.016
After 12 h (°C)	37.9 ± 0.9	37.4 ± 0.9	< 0.001	38.0 ± 1.0	37.4 ± 0.8	< 0.001
After 16 h (°C)	38.0 ± 1.0	37.4 ± 0.8	< 0.001	38.0 ± 0.9	37.3 ± 0.8	< 0.001
After 20 h (°C)	38.2 ± 0.9	37.4 ± 0.7	< 0.001	38.2 ± 0.6	37.4 ± 0.8	< 0.001
After 24 h (°C)	38.0 ± 0.6	37.4 ± 0.7	< 0.001	38.2 ± 0.6	37.5 ± 0.7	< 0.001
After 28 h (°C)	38.3 ± 1.0	37.5 ± 0.8	< 0.001	37.9 ± 0.7	37.4 ± 0.7	< 0.001
After 32 h (°C)	37.7 ± 1.0	37.0 ± 2.4	0.040	37.9 ± 1.0	37.2 ± 0.7	0.001
After 36 h (°C)	37.9 ± 0.9	37.1 ± 0.7	< 0.001	37.6 ± 0.7	37.1 ± 0.8	0.001
After 40 h (°C)	38.1 ± 0.9	37.1 ± 0.7	< 0.001	37.9 ± 0.9	37.0 ± 0.7	< 0.001
After 44 h (°C)	38.1 ± 0.8	37.1 ± 0.7	< 0.001	37.8 ± 0.8	37.1 ± 0.7	< 0.001

Continuous variables are presented as mean ± standard deviation

Categorical variables are presented as frequencies (%)

*ESR* Erythrocyte sedimentation rate, *CRP* C-reactive protein, *LDH* Lactate dehydrogenase, *AST* Aspartate aminotransferase, *ALT* Alanine aminotransferase, *WBC* White blood cell

### Model development for predicting RMPP-3 from the training cohort

Univariate logistic analysis identified that mean WBC count, percentage of neutrophils, absolute neutrophil count, percentage of lymphocytes, absolute lymphocyte count, platelets, CRP, LDH, radiologic grouping, and presence of pleural effusion were significantly associated with RMPP-3 grouping (*p*< 0.05). Using all significant variables from the univariate analysis, a conventional logistic model using stepwise procedure predicting RMPP-3 was created, which only selected four variables including platelets (odds ratio (OR) 0.991, *p*< 0.001), CRP (OR 1.014, *p*< 0.001), LDH (OR 1.006, *p*< 0.001), and radiologic grouping (*p*< 0.001) as significant components of the prediction model (shown in Table 3).

**Table 3:** Conventional logistic model using data of the training cohort: RMPP-3

	Univariate			Multivariate		
	Odds ratio	95% confidence interval	*p*-value	Odds ratio	95% confidence interval	*p*-value
Age (years)	1.046	(0.985, 1.110)	0.140			
Sex (female: male)	1.345	(0.922, 1.955)	0.125			
WBC × 10^3^/µL	1.000	(1.000, 1.000)	< 0.001			
Neutrophils (%)	1.024	(1.009, 1.039)	0.002			
Absolute neutrophil count × 10^3^/µL	1.000	(1.000, 1.000)	0.018			
Lymphocytes (%)	0.972	(0.955, 0.990)	0.002			
Absolute lymphocyte count × 10^3^/µL	0.999	(0.999, 1.000)	< 0.001			
Hemoglobin (g/dL)	0.946	(0.772, 1.159)	0.590			
Platelets × 10^3^/µL	0.991	(0.989, 0.994)	< 0.001	0.991	(0.988, 0.994)	< 0.001
ESR (mm/hr)	1.007	(0.997, 1.017)	0.179			
CRP (mg/L)	1.019	(1.013, 1.026)	< 0.001	1.014	(1.008, 1.021)	< 0.001
Procalcitonin (ng/mL)	0.973	(0.845, 1.120)	0.700			
LDH (IU/L)	1.004	(1.002, 1.007)	< 0.001	1.006	(1.003, 1.009)	< 0.001
AST (IU/L)	1.006	(0.998, 1.013)	0.126			
ALT (IU/L)	0.999	(0.995, 1.003)	0.563			
Concurrent respiratory virus	1.444	(0.919, 2.270)	0.111			
Oxygen requirement	0.766	(0.247, 2.379)	0.645			
Radiologic grouping			< 0.001			< 0.001
Group 1	–	–	–			
Group 2	2.786	(1.451, 5.352)	0.002	2.408	(1.209, 4.796)	0.012
Group 3	10.385	(5.379, 20.049)	< 0.001	8.080	(4.005, 16.302)	< 0.001
Group 4	91.385	(10.899, 766.257)	< 0.001	11.827	(1.181, 118.493)	0.036
Pleural effusion	4.191	(2.325, 7.552)	< 0.001			

*RMPP-3* Refractory Mycoplasma pneumoniae pneumonia with fever for ≥ 72 h, *ESR* Erythrocyte sedimentation rate, *CRP* C-reactive protein, *LDH* Lactate dehydrogenase, *AST* Aspartate aminotransferase, *ALT* Alanine aminotransferase

### Model development for predicting RMPP-5 from the training cohort

Univariate logistic analysis identified sex, mean WBC count, percentage of neutrophils, percentage of lymphocytes, absolute lymphocyte count, platelets, CRP, LDH, radiologic grouping, pleural effusion, all fever profiles, and 12 sequential body temperatures as significantly associated with RMPP-5 grouping (*p*< 0.05). Using all significant variables from the univariate analysis, a conventional logistic model using stepwise procedure predicting RMPP-5 was created, which only selected three variables including radiologic grouping (*p*< 0.001), the lowest temperature (OR 6.494, *p*< 0.001), and the frequency of peak fever within 48 h (OR 1.603, *p*< 0.001) as significant components of the prediction model (shown in Table 4).

**Table 4:** Conventional logistic model using data of the training cohort: RMPP-5

	Univariate			Multivariate		
	Odds ratio	95% confidence interval	*p*-value	Odds ratio	95% confidence interval	*p*-value
Age (years)	1.046	(0.963, 1.138)	0.287			
Sex (female: male)	0.500	(0.292, 0.857)	0.012			
WBC × 10^3^/µL	1.000	(1.000, 1.000)	0.017			
Neutrophils (%)	1.046	(1.022, 1.071)	< 0.001			
Absolute neutrophil count × 10^3^/µL	1.000	(1.000, 1.000)	0.292			
Lymphocytes (%)	0.951	(0.924, 0.978)	< 0.001			
Absolute lymphocyte count × 10^3^/µL	0.999	(0.998, 0.999)	< 0.001			
Hemoglobin (g/dL)	0.950	(0.713, 1.266)	0.728			
Platelet × 10^3^/µL	0.982	(0.977, 0.987)	< 0.001			
ESR (mm/hr)	0.994	(0.979, 1.008)	0.391			
CRP (mg/L)	1.015	(1.010, 1.021)	< 0.001			
Procalcitonin (ng/mL)	1.056	(0.895, 1.245)	0.518			
LDH (IU/L)	1.004	(1.002, 1.006)	0.001			
AST (IU/L)	1.003	(0.997, 1.009)	0.355			
ALT (IU/L)	0.996	(0.986, 1.006)	0.420			
Concurrent respiratory virus	0.624	(0.323, 1.207)	0.161			
Oxygen requirement	3.111	(0.930, 10.412)	0.066			
Radiologic grouping			< 0.001			0.007
Group 1	–	–	–	–	–	–
Group 2	3.464	(0.998, 12.020)	< 0.001	1.693	(0.447, 6.403)	0.438
Group 3	9.778	(2.919, 32.757)	< 0.001	3.457	(0.942, 12.681)	0.061
Group 4	102.667	(20.318, 518.780)	< 0.001	13.947	(2.260, 86.077)	0.005
Pleural effusion	4.884	(2.578, 9.252)	< 0.001			
Fever profiles						
Highest temperature (°C)	5.424	(3.501, 8.403)	< 0.001			
Lowest temperature (°C)	54.127	(19.031, 153.945)	< 0.001	6.494	(2.102, 20.068)	0.001
Frequency of fever of > 39 °C within 48 h (*n*)	1.691	(1.494, 1.914)	< 0.001			
Frequency of fever of > 40 °C within 48 h (*n*)	3.230	(2.073, 5.033)	< 0.001			
Frequency of peak fever within 24 h (*n*)	2.676	(2.097, 3.413)	< 0.001			
Frequency of peak fever within 48 h (*n*)	1.872	(1.620, 2.162)	< 0.001	1.603	(1.361, 1.887)	0.001
12 sequential body temperatures						
Initial (°C)	2.017	(1.532, 2.654)	< 0.001			
After 4 h (°C)	1.988	(1.467, 2.695)	< 0.001			
After 8 h (°C)	1.852	(1.386, 2.474)	< 0.001			
After 12 h (°C)	1.960	(1.471, 2.610)	< 0.001			
After 16 h (°C)	2.134	(1.606, 2.834)	< 0.001			
After 20 h (°C)	3.740	(2.611, 5.358)	< 0.001			
After 24 h (°C)	3.138	(2.158, 4.562)	< 0.001			
After 28 h (°C)	2.926	(2.142, 3.996)	< 0.001			
After 32 h (°C)	1.930	(1.425, 2.615)	< 0.001			
After 36 h (°C)	2.929	(2.122, 4.043)	< 0.001			
After 40 h (°C)	3.446	(2.419, 4.908)	< 0.001			
After 44 h (°C)	3.873	(2.720, 5.515)	< 0.001			

*RMPP-5* Refractory Mycoplasma pneumoniae pneumonia with fever for ≥ 120 h, *ESR* Erythrocyte sedimentation rate, *CRP* C-reactive protein, *LDH* Lactate dehydrogenase, *AST* Aspartate aminotransferase, *ALT* Alanine aminotransferase

**Fig. 2:**
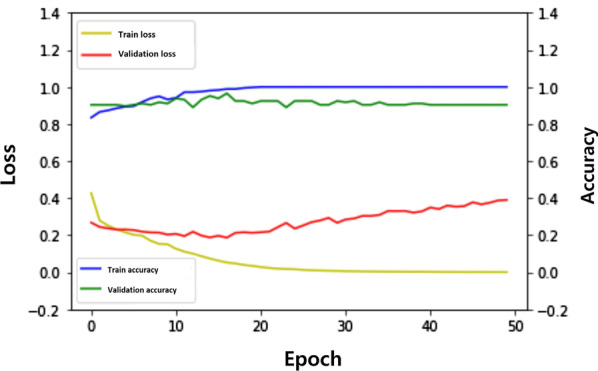
Loss and accuracy of the deep neural network (DNN) model

Including two hidden layers (128 neurons in the first layer and 64 neurons in the second layer), a DNN model was created using 12 sequential body temperatures. The validation loss and validation accuracy of the DNN model were 0.1807 and 0.9172, respectively (epoch = 15, Fig. 2).

### Prediction of RMPP-3 in the test cohort

**Fig. 3:**
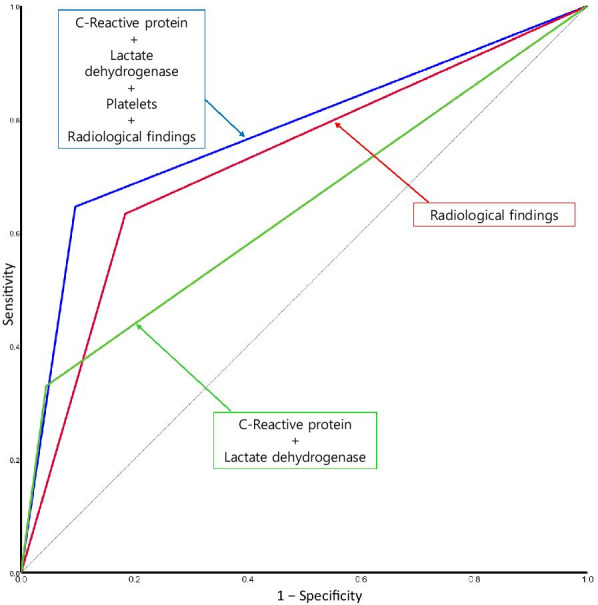
A receiver operating characteristic (ROC) curve for the prediction of RMPP-3

The performance of conventional logistic models predicting RMPP-3 is compared in Table 5. Among the prediction models using individual variables, the prediction model using radiologic grouping showed the best values (area under the curve (AUC) 0.725, sensitivity 63.3%, and specificity 81.6%). The prediction power of the model was further increased by the addition of platelets, CRP, and LDH (AUC 0.775, sensitivity 64.6%, and specificity 90.4%) as laboratory variables (Table 5, Fig. 3).

**Table 5:** Prediction of RMPP-3 (fever > 72 h) in the test cohort

Prediction method	Used variables	*p*-value	AUC area (95% CI)	Sensitivity (%)	Specificity (%)	PPV (Precision) (%)	NPV (%)	Overall accuracy (%)	Youden Index
Conventional logistic model	CRP, LDH	< 0.001	0.642 (0.562, 0.723)	32.9	95.2	81.3	69.4	71.2	0.281
Conventional logistic model	Radiologic grouping	< 0.001	0.725 (0.651, 0.798)	63.3	81.6	66.7	79.3	74.9	0.449
Conventional logistic model	Platelets, CRP, LDH, radiologic grouping	< 0.001	0.775 (0.704, 0.846)	64.6	90.4	79.7	81.5	80.9	0.550

*RMPP-3* Refractory Mycoplasma pneumoniae pneumonia with fever for ≥ 72 h, *AUC* Area under the curve, *CRP* C-reactive protein, *LDH* Lactate dehydrogenase, *PPV* Positive predictive value, *NPV* Negative predictive value

### Prediction of RMPP-5 in the test cohort

**Fig. 4:**
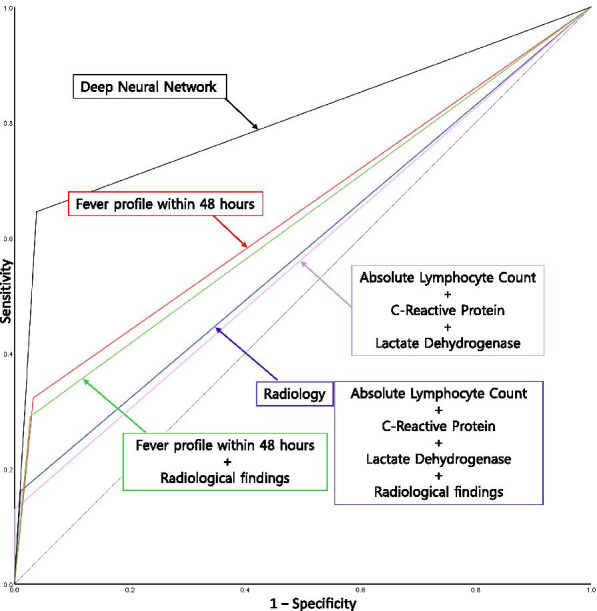
A receiver operating characteristic (ROC) curve for the prediction of RMPP-5

The performance of conventional logistic models predicting RMPP-5 is compared in Table 6. While conventional logistic models using only radiological grouping did not show significant predictive power in the test cohort, prediction models using the fever profiles (lowest temperature and frequencies of peak fever) showed significant predictive power, although with a sensitivity of 32.3% and a specificity of 96.7% in the test cohort (AUC 0.645, *p* = 0.010). However, the DNN model using data of 12 sequential body temperatures demonstrated a better and significant outcome (sensitivity 64.5% and specificity 96.2%, AUC 0.803, *p*< 0.001) (Fig. 4).

**Table 6:** Prediction of RMPP-5 (fever > 120 h) in the test cohort

Prediction method	Used variables	*p*-value	AUC area (95% CI)	Sensitivity (%)	Specificity (%)	PPV (Precision) (%)	NPV (%)	Overall accuracy (%)	Youden Index
Conventional logistic model	ALC, CRP, LDH	0.251	0.565 (0.447, 0.682)	12.9	100.0	100.0	87.1	87.4	0.129
Conventional logistic model	Radiologic grouping	0.181	0.575 (0.457, 0.693)	16.1	98.9	71.4	87.5	87.0	0.150
Conventional logistic model	ALC, CRP, LDH, Radiologic grouping	0.181	0.575 (0.457, 0.693)	16.1	98.9	71.4	87.5	87.0	0.150
Conventional logistic model	Fever profiles*	0.010	0.645 (0.525, 0.765)	32.3	96.7	62.5	89.4	87.4	0.290
Conventional logistic model	Fever profiles*, Radiologic grouping	0.019	0.632 (0.512, 0.751)	29.0	97.3	64.3	89.1	87.4	0.263
Deep neural network	12 sequential fever data	< 0.001	0.803 (0.699, 0.908)	64.5	96.2	74.1	94.1	91.6	0.607

*RMPP-5* Refractory Mycoplasma pneumoniae pneumonia with fever for ≥ 120 h, *AUC* Area under the curve, *ALC* Absolute lymphocyte count, *CRP* C-reactive protein, *LDH* Lactate dehydrogenase, *PPV* Positive predictive value, *NPV* Negative predictive value

^*^Fever profiles: highest body temperature, lowest body temperature, frequency of peak fever over 39 °C, frequency of peak fever over 40 °C, and total frequency of peak fever

## Discussion

To prevent the progression of MP pneumonia resulting in severe and prolonged clinical course, early recognition and timely treatment is important for patients who display clinical and radiological aggravation during macrolide therapy [8, 16, 23, 24]. To our knowledge, this is the first study to demonstrate a prediction model for refractory MP pneumonia based on readily accessible sequential fever data in addition to clinical, laboratory, and radiologic variables at admission. For prediction of RMPP-3, a conventional logistic model using only radiologic grouping showed increased sensitivity (63.3%) than the model using laboratory values, including CRP and LDH. Adding laboratory values in the prediction model using radiologic grouping did not meaningfully contribute to an increase in sensitivity (64.6%). For the prediction of RMPP-5, laboratory values and radiologic grouping showed lower sensitivities ranging from 12.9 to 16.1%. However, prediction models using the predefined fever profiles showed significantly increased sensitivity for predicting RMPP-5, and neural network models using 12 sequential fever data showed a greatly increased sensitivity of 64.5%. Predicting high-risk patients for refractory MP pneumonia would enable physicians to calibrate their expectations of progression in these patients and to provide earlier alternative treatment.

Several studies have tried to identify predictors for refractory MP pneumonia and have suggested individual cut-off values of inflammatory markers, namely CRP, LDH, and ferritin or cytokines, such as IL-6, IL-8, IL-10, IL-18, and interferon-gamma [15–17, 19, 23]. However, the application of these findings in clinical practice is limited by lower prediction power or accessibility of the tests. Although it is plausible that increased inflammatory cytokines are related to the severity of MP pneumonia, serum cytokine assays are mostly limited for research purposes and are not routinely measured. Bronchoscopy and bronchoalveolar lavage studies are useful tools not only for identifying the causative organism but also for the removal of mucosal plugs in severe pneumonia, but are generally performed for a small proportion of MP pneumonia cases. The requirement of sedation, the necessity of special equipment, and the need for an experienced bronchoscopist limit their accessibility.

A study using CRP value of 16.5 mg/L as the cut-off value showed a sensitivity of 74.7% and a specificity of 77.2% for predicting refractory MP pneumonia [18]. However, our prediction model created using the CRP level of the training cohort showed a sensitivity of 32.9% for the prediction of RMPP-3 in the test cohort even when it was combined with the LDH level (Table 5). For the prediction of RMPP-5, our prediction model using the CRP level showed a lower sensitivity of 12.9% even when used in combination with ALC and LDH levels (Table 6). Previous prediction models, created without validation, are inevitably vulnerable to model overfitting, resulting from institutional selection bias, which limits their clinical use. Therefore, a reasonable prediction model should undergo internal validation by a separate test cohort or external validation using data from another institution. Thus, it is understandable that previously identified laboratory markers such as CRP and LDH showed lower sensitivities (below 30%) for predicting RMPP-3 and RMPP-5 in our cohorts (Tables 5, 6). Such low sensitivities limit their clinical application for the timely detection of refractory MP pneumonia. To overcome such bias, our 716 enrolled patients were divided into training and test datasets for internal validation, which prevented overfitting and created a reasonable prediction model.

For prediction of RMPP-3, according to a previous study, initial radiologic grouping was the most prominent predictor [25]. While the underlying mechanisms are still unclear, the pattern of pulmonary lesions in MP infection is reported to be influenced by the characteristics of host cell-mediated immunity [26, 27]. Thus, radiological evidence of lung involvement is consistent with the strong host immune response in RMPP.

Both initial laboratory values and radiologic grouping showed limited prediction power for the prediction of RMPP-5. However, we tried to predict RMPP using initially available data and focused on the fever data during the initial 48-h period. Inflammatory cytokines involved in the immunopathogenesis of MP infection are reported to be increased in RMPP [3, 20, 28]. Since these cytokines act as endogenous pyrogens that play a pivotal role in inducing fever response, their levels are associated with core body temperature [29]. Although initial single time-point data were limited for predicting RMPP-5, the prediction model using predefined fever profiles showed a two-fold increase in sensitivity (16.1% to 32.3%), and the DNN model using all 12 sequential fever data within 48 h showed a four-fold increase in sensitivity (64.5%) for predicting RMPP-5. Theoretically, DNN is a black-box approach, and the causes of superior prediction power of the DNN model cannot be identified. However, the greatly increased sensitivity for predicting RMPP-5 with the DNN model using only the initial 48-h fever data is noteworthy.

The major purpose of our grouping that included RMPP-3 and RMPP-5 was to evaluate the prediction power of the statistical model at two separate time points, to compare their prediction power, and to infer the causes for the difference. The prediction power of our statistical models for the later event (RMPP-5) was considerably lower than that for the early event (RMPP-3). Evaluating the model prediction power at separate time points enabled us to trace the changing trends in the variables of the prediction models at different time points. We identified fever profiles and radiologic grading as the most effective predictors that have superior prediction power for the ‘later event’ (RMPP-5).

The main limitation of our study is its retrospective design based on a limited number of inpatients from a single center, which might have introduced a selection bias. However, our prediction models underwent internal validation. Prediction models were created only from the data in the training cohort, and their prediction power was estimated in the test cohort, which was not used for model development. Nevertheless, external validation of our model in a prospective, large-scale cohort is needed for validating our results. Second, a possibility of under-diagnosis and over-diagnosis in MPP exists because of false negative IgM antibodies in the early stage or persistent IgM antibodies in convalescent patients with recent infection. We attempted to minimize these misdiagnoses through our strict exclusion criteria. Third, prediction models were not developed using tests, namely cytokines or FOB, which were reported to be significant. We especially focused on the accessibility of the tests, and those tests were not considered useful in usual clinical practice. Lastly, data on macrolide resistance were not included. Although febrile days during macrolide administration were reported to be greater in macrolide-resistant patients (3.5–4.0 days vs. 1.0–1.5 days) [9, 30], prolonged fever in RMPP patients may not imply macrolide resistance because fever might have resolved spontaneously in some macrolide-resistant patients. The clinical efficacy of macrolide for treating MP infection may not only reflect its direct antimicrobial activity but also reflect its anti-inflammatory effects [31].

Development of tests based on data obtained from routine examination of vital signs and its integration into the clinical workflow can be more effective than utilizing new tests that are less verified and less accessible. Further studies utilizing such potential data are needed for improving the prediction power.

## Conclusion

In summary, our study showed that for prediction of RMPP-3, a conventional logistic model using only radiologic grouping showed a favorable predictive power than the model using initial laboratory values. In contrast, RMPP-5 could not be effectively predicted using the initial laboratory and radiologic data, which were previously reported to be significantly predictive. However, the prediction models using predefined fever profiles showed a two-fold increase in sensitivity (16.1–32.3%), and the DNN model using all 12 sequential fever data within 48 h showed a four-fold increase in sensitivity (64.5%). Further studies using more advanced mathematical models based on easily accessible large-sized clinical data are anticipated to be helpful for predicting RMPP.

## Data Availability

The datasets used and/or analysed during the current study are available from the corresponding author on reasonable request.

## Abbreviations

CBC: Complete blood count
CRP: C-reactive protein
DNN: Deep neural network
ESR: Erythrocyte sedimentation rate
LDH: Lactate dehydrogenase
MP: *Mycoplasma pneumoniae*
MPP: *Mycoplasma pneumoniae* Pneumonia
NPA: Nasopharyngeal aspirate/swab
RMPP: Refractory MPP
ROC: Receiver operating characteristic
